# Predictive value of 25-hydroxyvitamin D level in patients with coronary artery disease: A meta-analysis

**DOI:** 10.3389/fnut.2022.984487

**Published:** 2022-08-10

**Authors:** Hailing Zhang, Pei Wang, Yu Jie, Yimeng Sun, Xiaoyan Wang, Yu Fan

**Affiliations:** ^1^Center of Clinical Medical Research, The Affiliated Suqian First People's Hospital of Xuzhou Medical University, Suqian, China; ^2^Institute of Molecular Biology and Translational Medicine, The Affiliated People's Hospital, Jiangsu University, Zhenjiang, China

**Keywords:** 25-hydroxyvitamin D, coronary artery disease, major adverse cardiovascular events, mortality, meta-analysis

## Abstract

**Background:**

A consensus has not been made about the predictive value of blood vitamin D level in patients with coronary artery disease (CAD). This meta-analysis aimed to assess the association between blood 25-hydroxyvitamin D level and adverse outcomes in patients with CAD.

**Methods:**

Two independent authors searched the articles indexed in PubMed and Embase databases until June 28, 2022. Cohort studies or *post-hoc* analysis randomized trials evaluating the value of 25-hydroxyvitamin D level in predicting cardiovascular or all-cause mortality, and major adverse cardiovascular events ([MACEs] including death, non-fatal myocardial infarction, heart failure, revascularization, stroke, etc.) were included.

**Results:**

The literature search identified 13 eligible studies for our analysis, including 17,892 patients with CAD. Meta-analysis showed that the pooled adjusted risk ratio (RR) was 1.60 (95% confidence intervals [CI] 1.35–1.89) for all-cause mortality, 1.48 (95% CI 1.28–1.71) for cardiovascular mortality, and 1.33 (95% CI 1.18–1.49) for MACEs. Leave-out one study sensitivity analysis suggested that the predictive values of blood 25-hydroxyvitamin D level were reliable.

**Conclusions:**

Low blood 25-hydroxyvitamin D level is possibly an independent predictor of cardiovascular or all-cause mortality and MACEs in patients with CAD. Baseline 25-hydroxyvitamin D level may provide useful information in CAD patients.

## Introduction

Coronary artery disease (CAD) is the most common type of heart disease worldwide, which can be manifested as stable ischemic heart disease or acute coronary syndrome (ACS). Despite the improvement in medical therapy and surgical revascularization, CAD remains a major determinant of morbidity and premature death (1). A more aggressive secondary prevention can be implemented by employing early risk stratification for cardiovascular events and death in patients with CAD.

Biomarkers play an important role in risk stratification and management of CAD (2, 3). Vitamin D is a hormone precursor that maintains calcium homeostasis. The blood level of 25-hydroxyvitamin D is identified as the best estimation of vitamin D state (4). Increasing attention has been focused on the effect of vitamin D on the management of cardiovascular disease (5). Vitamin D deficiency or insufficiency is prevalent in patients with CAD (6–8). Low blood 25-hydroxyvitamin D level is emerging as a predictive biomarker for patients with CAD (9–13). However, inconsistent findings (14–17) have been recorded on the predictive value of Vitamin D deficiency in these patients.

No previous meta-analysis has systematically assessed the predictive association of Vitamin D deficiency with adverse outcomes in patients with CAD. Therefore, the current meta-analysis aimed to evaluate the predictive value of blood 25-hydroxyvitamin D level of patients with CAD in terms of cardiovascular death, all-cause mortality, and cardiovascular events.

## Methods

### Search strategy

The current meta-analysis was carried out under the Preferred Reporting Items for Systematic Reviews and Meta-analysis guideline (18). Two independent authors identified the eligible studies indexed in PubMed and Embase databases using the following combination of items: (“vitamin D” OR “25-hydroxyvitamin D”) AND (“coronary artery disease” OR “coronary heart disease” OR “ischemic heart disease” OR “ischaemic heart disease” OR “acute coronary syndrome” OR “myocardial infarction” OR “angina”) AND (“death” OR “mortality” OR “cardiovascular event”) AND (“follow-up” OR “follow up” (Supplementary Text S1 in Supplementary material). The final updated search was conducted on June 28, 2022. References of pertinent articles were manually scanned to identify potentially eligible studies. To minimize publication bias, we also reviewed the ClinicalTrials.gov and full-text database of Chinese Excellent Doctoral and Master's Dissertations to identify any gray and unpublished literature.

### Study selection

The inclusion criteria are as follows: (1) population: patients were with CAD; (2) exposure: blood 25-hydroxyvitamin D level at baseline; (3) comparison: patients with the bottom vs. reference top 25-hydroxyvitamin D level; (4) outcome measures: cardiovascular or all-cause mortality, and major adverse cardiovascular events ([MACEs] including death, non-fatal myocardial infarction, heart failure, revascularization, stroke, etc.); (5) reported multivariable adjusted risk estimate for the above mentioned outcomes; and (6) study design: retrospective or prospective cohort studies or *post-hoc* analysis randomized trials. When multiple articles were obtained from the same population, we selected the publication with the longest follow-up. The exclusion criteria are as follows: (1) studies reported the in-hospital outcomes; (2) studies provided risk summary by continuous 25-hydroxyvitamin D level; and (3) cross-sectional study or meeting abstract.

### Data extraction and quality assessment

The following data was abstracted by two authors independently: last name of the first author, publication year, origin of study, study design, subtype of CAD, number of patients, gender distribution, age of patients at enrollment, length of follow-up, cutoff value of vitamin D deficiency, definition of MACEs, endpoints, fully adjusted risk summary, and confounders included in the fully adjusted models. Two authors independently assessed the study quality according to the Newcastle-Ottawa Scale (NOS) for cohort studies (maximum score of 9 points) (19). Studies with NOS point ≥ 7 indicated high methodological quality. Any discrepancies were settled by discussing with a third author (Y Fan) to reach consensus.

### Statistical analysis

All data were analyzed using STATA 12.0 (STATA Corp LP, College Station, TX, USA). To evaluate the association between blood 25-hydroxyvitamin D level and adverse outcomes, we poled the most fully adjusted risk ratios (RR) and 95% confidence intervals (CI) with the bottom vs. the reference top category of 25-hydroxyvitamin D level. Heterogeneity between studies was determined using the Cochran's Q statistic (p <0.10 was considered significant) and the *I*^2^ statistic (*I*^2^≥ 50% was considered significant). A random effect model was used for data analysis in the presence of statistically significant heterogeneity. A fixed-effect model was used in the absence of significant heterogeneity. To test the credibility of the pooling results, we conducted a leave-out one study sensitivity analysis to recalculate the risk estimates. Subgroup analyses were conducted to investigate the effect of the types of CAD, sample sizes, publication time, and length of follow-up. Begg's test, Egger's test, and funnel plot were used to investigate the publication bias. The certainty of evidence was summarized *via* the GRADE analysis.

## Results

### Search results and study characteristics

**Figure 1:**
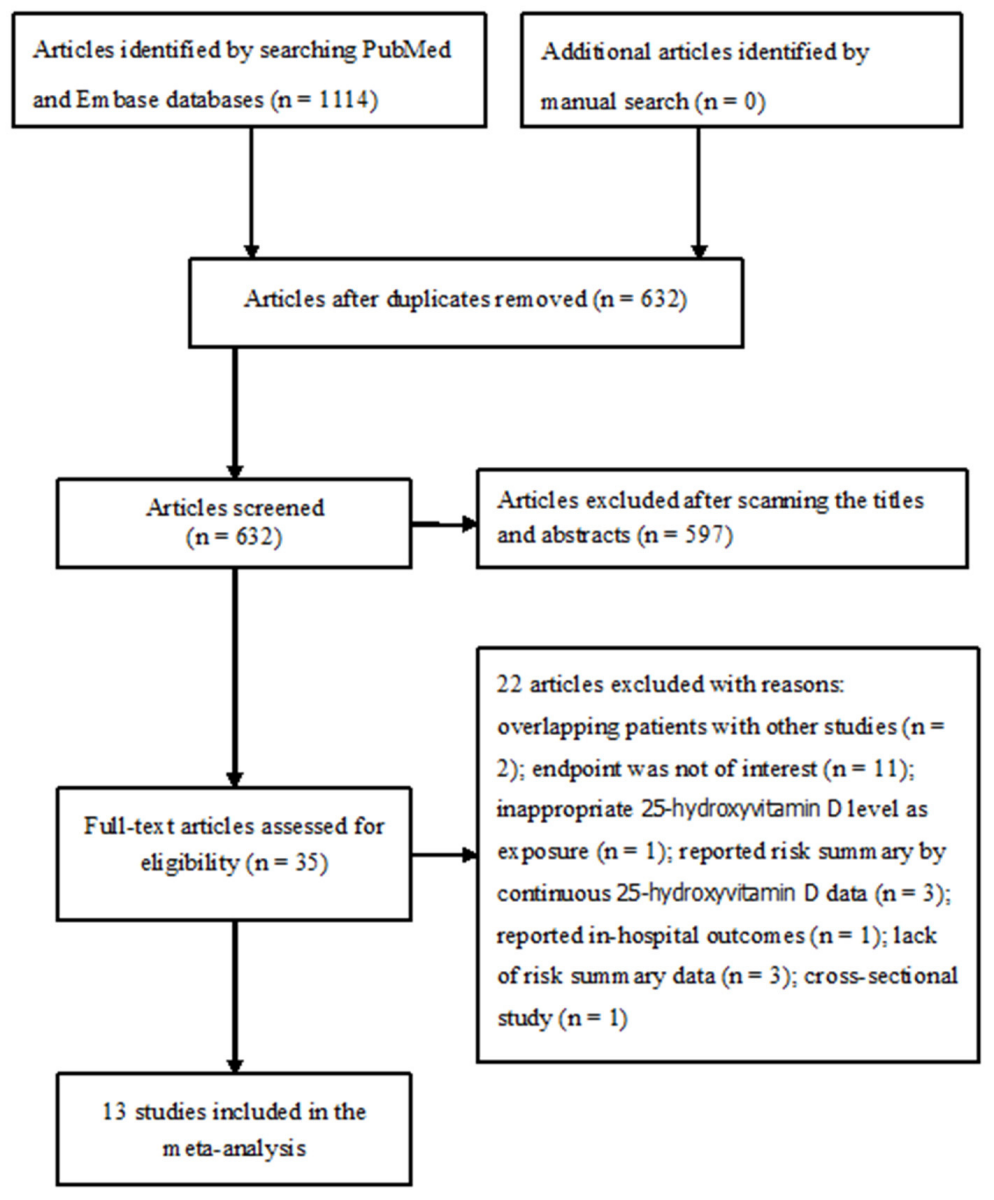
Flow chart of the study selection process.

A total of 1,114 records were identified from initial electronic database search. After excluding duplicates, 632 records were left. After reading the titles and abstracts, 597 obviously unrelated records were excluded. Thirty-five articles were retrieved for full-text assessment, and 13 studies (9–17, 20–23) satisfied the inclusion criteria (Figure 1).

The main features of the included studies are presented in Table 1. These included studies were published between 2010 and 2021. All articles adopted the prospective designs. Four studies (10, 11, 17, 21) included patients with ACS, one study (22) enrolled patients with post-acute myocardial infarction (AMI), one study (13) included stable angina patients, and others did not report the specific type of CAD. Sample sizes ranged from 252 to 4,114, with a total of 17,892 patients with CAD. The length of follow-up varied from 12 months to 11.9 years. According to the NOS criteria, all studies were deemed as high methodological quality (Supplementary Table S1).

**Table 1:** Main characteristic of the included studies.

**Author/ year**	**Region**	**Patients (% male)**	**Age (years)**	**Cutoff value of 25(OH) D level**	**Definition of MACEs**	**Outcome measures HR/RR (95% CI)**	**Follow-up**	**Adjusted variables**
Grandi et al. (14)	Germany	Stable CAD 1,125 (84.4)	30–70	<15 *vs*. >30 ng/mL	Non-fatal MI, ischemic stroke, or CV death	Total death 0.94 (0.39–2.26); MACEs 0.83 (0.37–1.86)	8.1 years	Age, sex, season, smoking, hypertension, DM, BMI, TG, LDL, HDL, TC, number of affected vessels, previous MI, creatinine clearance; treatment, CRP
Lerchbaum et al. (9)	Austria	CAD 2,069 (100)	54–74	<11.2 *vs*. ≥11.2 ng/mL	—	Total death; 1.77 (1·47–2.13); CV death; 1.24 (0.96–1.60)	7.7 years	Age, BMI, active smoking, physical activity, DM, CRP, prevalent CAD, serum calcium, parathyroid hormone.
Ng et al. (10)	UK	AMI 1,259 (72)	65.7 ± 12.8	<7.3 *vs*. >20 ng/mL	Death, HF admission, recurrent MI	Total death; 1.25 (0.75–2.08); MACEs; 1.61 (1.15–2.27)	1.5 years	Age, sex, previous MI, hypertension, DM, Killip class, eGFR, NTproBNP, smoking, electrocardiogram ST change
Siasos et al. (15)	Greece	CAD 252 (91)	62 ± 11	<30 *vs*. ≥30 ng/mL	CV death, non-fatal MI, or stroke, admission for cardiovascular causes	MACEs; 7.24 (0.99–53.5)	1.25 years	Age, sex, kidney function, dyslipidemia, hypertension, DM, smoking, obesity, severity of CAD
Welles et al. (16)	USA	CAD 946 (81)	65.4 ± 11	<30 *vs*. ≥20 ng/mL	CV death, HF, MI, stroke	Total death; 1.18 (0.92–1.52); CV death; 1.13 (0.76–1.70); MACEs; 1.11 (0.85–1.44)	8.0 years	Age, sex, race/ethnicity, season of blood draw, college graduation, tobacco use, multivitamin use, physical activity, DM, hypertension, depression, BMI, SBP, DBP, hemoglobin A1c, TG, HDL, CRP, phosphorus, parathyroid hormone, fibroblast growth factor 23
De Metrio et al. (11)	Italy	ACS 814 (72)	67 ± 12	<9.0 *vs*. >22 ng/mL	Death, arrhythmias, cardiogenic shock, AKI, major bleeding, APE	Total death; 2.51 (1.35–4.65); MACEs; 1.85 (1.25–2.75)	1.0 year	Age, BMI, DM, LVEF, creatinine, HDL, TC, TG
Naesgaard et al. (17)	Norway	ACS 871 (61)	69.6 ± 14.4	Quartiles 1 *vs*. Quartiles 4	—	Total death; 1.27 (0.92–1.75); CV death; 1.20 (0.58–2.50)	7.0 years	Age, sex, smoking, hypertension, BMI, index diagnosis, DM, chronic HF, previous CAD, hypercholesterolemia, use of statins, troponin-T, eGFR, hsCRP, BNP, β-blockers
Gerling et al. (12)	Canada	CAD 2,975 (60)	63.6 ± 12	≤ 40.2 *vs*. ≥ 91.8 nmol/L	—	Total death; 1.84 (1.36–2.50)	5.8 years	Age, sex, BMI, smoking, renal disease, hypertension, hyperlipidemia, type 2 DM, family history of heart disease, prior MI, congestive HF
Yu et al. (20)	China	CAD 1,387 (65.1)	40–85	≤ 2.11 *vs*. ≥4.88 ng/mL	—	Total death; 1.36 (0.88–2.12); CV death; 1.49 (0.87–2.56)	6.7 years	Age, sex, BMI, smoking, DM, SBP, TC, HDL, extent of CAD, acute CAD, coronary revascularization, use of statins, ACEI/ARB, β-blockers, season of blood-drawing, physical activity, eGFR, calcium, parathyroid hormone, and CRP
Degerud et al. (13)	Norway	Stable angina 4,114 (71.9)	61.8 ± 10.4	≤ 13.6 *vs*. > 13.6–32.1 ng/mL	—	Total death; 1.94 (1.66–2.27); CV death; 1.87 (1.49–2.36)	11.9 years	Age, sex, study site, smoking, BMI, SBP, eGFR
Beska et al. (21)	UK	NSTE-ACS 294 (61.9)	80.5 ± 4.8	<9.45 *vs*. ≥9.45 ng/mL	Death, ACS, stroke, revascularization, major bleeding	MACEs; 1.20 (0.72–2.0)	1.0 year	Age, sex, time of blood collection, hypertension, previous MI, congestive HF, Charlson index, Rockwood Frailty Score, hemoglobin, hs-CRP, vitamin D supplementation
Aleksova et al. (22)	Italy	Post-MI 1,081 (70.9)	66.7 ± 11.5	≤ 20 *vs*. >20 ng/mL	Death, angina/MI, HF	MACEs; 1.3 (1.04–1.64)	2.2 years	Age, sex, season, multivessel disease, previous coronary events/revascularization, CRP, eGFR, LVEF, ACEI/ARB, β-blockers
Verdoia et al. (23)	Italy	CAD 705 (77.6)	67.3 ± 10.8	<12.7 *vs*. ≥21.6 ng/mL	Death, MI, TVR	Total death; 3.6 (1.43–8.9); CV death; 4.28 (0.57–32); MACEs; 1.32 (1.07–1.63)	2.7 years	Age, sex, DM, CKD

HR, hazard ratio; RR, risk ratio; CI, confidence intervals; HF, heart failure; 25(OH) D, 25-hydroxyvitamin D; BMI, body mass index; DM, diabetes mellitus; LVEF, left ventricular ejection fraction; SBP, systolic blood pressure; DBP, diastolic blood pressure; BNP, B-type natriuretic peptide; NT-proBNP, N-terminal prohormone brain natriuretic peptide; LDL, low density lipoprotein cholesterol; HDL, high-density lipoprotein; TC, total cholesterol; TG, triglycerides; MI, myocardial infarction; AF, atrial fibrillation; eGFR, estimated glomerular filtration rate; CKD, chronic kidney disease; CAD, coronary artery disease; ACS, acute coronary syndrome; TVR, target vessel revascularization; APE, acute pulmonary edema; AKI, acute kidney injury; CRP, C-reactive protein; hs-CRP, high sensitivity C-reactive protein; ACEI, angiotensin converting enzyme inhibitors; ARB, angiotensin receptor blockers.

### All-cause mortality

**Figure 2:**
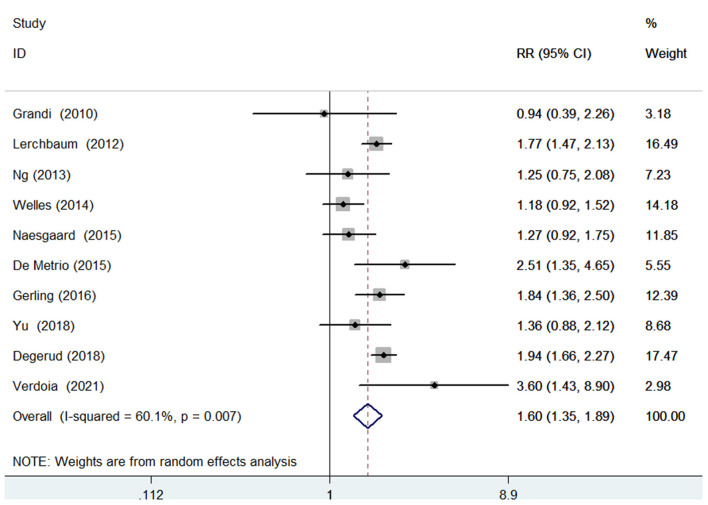
Forest plots showing pooled RR with 95% CI of all-cause mortality for the bottom vs. the reference top category of 25-hydroxyvitamin D level.

Ten studies (9–14, 16, 17, 20, 23) investigated the value of 25-hydroxyvitamin D level in predicting all-cause mortality. Figure 2 provides a pooling risk summary of the association between 25-hydroxyvitamin D level and all-cause mortality. Under a random effect model meta-analysis, the pooled adjusted RR of all-cause mortality was 1.60 (95% CI 1.35–1.89) for the bottom vs. the reference top category of 25-hydroxyvitamin D level, having significant heterogeneity (*I*^2^ =60.1%; *p* = 0.007). Leave-out one study sensitivity analysis did not alter the statistical significance of the original risk estimate. In addition, the value of blood 25-hydroxyvitamin D level in predicting all-cause mortality was consistently found in each named subgroup (Table 2). Publication bias was not detected in this outcome according to the Begg's test (*p* = 1.000), Egger's test (*p* = 0.567), and symmetrical funnel plot (Supplementary Figure S1).

**Table 2:** Results of subgroup analysis on all-cause mortality.

**Subgroup**	**Number of studies**	**Pooled RR**	**95% CI**	**Heterogeneity between studies**
Types of CAD				
ACS	3	1.49	1.02–2.18	p=0.138; I^2^ = 49.5%
All CAD	7	1.64	1.35–1.99	p=0.011; I^2^ = 63.9%
Follow-up duration				
≥3 years	7	1.54	1.29–1.84	p=0.010; I^2^ =64.4%
<3 years	3	2.08	1.12–3.86	p=0.073; I^2^ =61.8%
Sample sizes				
≥1000	6	1.74	1.52–1.99	p=0.258; I^2^ =23.4%
<1000	4	1.62	1.10–2.40	p=0.023; I^2^ = 68.5%
Publication time				
Before 2015	6	1.43	1.14–1.81	p=0.037; I^2^ =57.7%
				
After 2015	4	1.86	1.53–2.25	p=0.241; I^2^ =28.5%

CAD, coronary artery disease; ACS, acute coronary syndrome; RR, hazard ratio; CI, confidence intervals.

### Cardiovascular mortality

**Figure 3:**
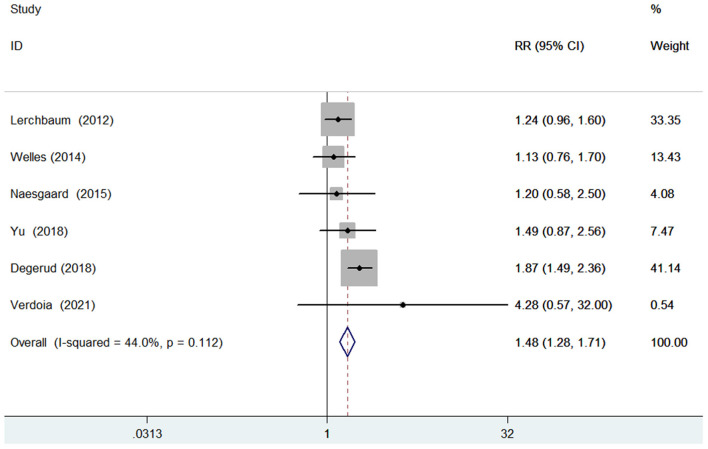
Forest plots showing pooled RR with 95% CI of cardiovascular mortality for the bottom vs. the reference top category of 25-hydroxyvitamin D level.

Six studies (9, 13, 16, 17, 20, 23) evaluated the value of 25-hydroxyvitamin D level in predicting cardiovascular mortality. Figure 3 shows a pooling risk estimate of the association between 25-hydroxyvitamin D level and cardiovascular mortality. Based on fixed-effect model meta-analysis, the pooled adjusted RR of cardiovascular mortality was 1.48 (95% CI 1.28–1.71) for the bottom vs. the reference top category of 25-hydroxyvitamin D level, and no significant heterogeneity was observed between studies (*I*^2^ = 44.0%; *p* = 0.112). Sensitivity analysis confirmed the robustness of the originally pooling risk estimate. The Begg's test (*p* = 1.000), Egger's test (*p* = 0.567), and symmetrical funnel plot (Supplemental Figure S2) suggested a low likelihood of publication bias.

### Major adverse cardiovascular events

**Figure 4:**
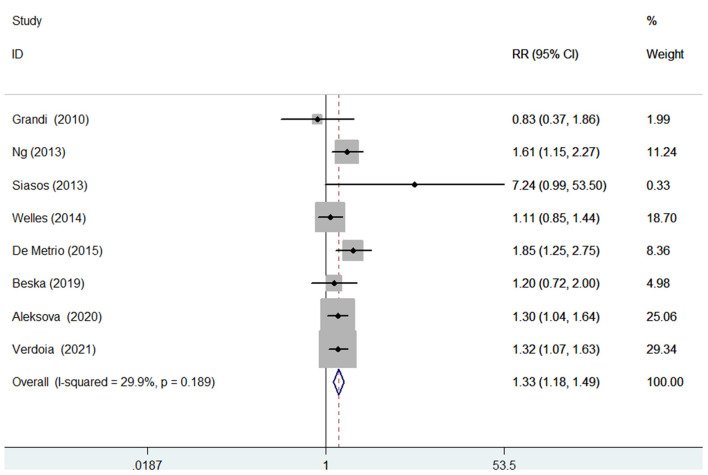
Forest plots showing pooled RR with 95% CI of major adverse cardiovascular events for the bottom vs. the reference top category of 25-hydroxyvitamin D level.

Seven studies (10, 11, 14–16, 21–23) evaluated the value of 25-hydroxyvitamin D level in predicting MACEs. Figure 4 provides a pooling risk estimate of the association between d-dimer level and MACEs. A fixed-effect model meta-analysis suggested that the pooled adjusted RR of MACEs was 1.33 (95% CI 1.18–1.49) for the bottom vs. the reference top category of 25-hydroxyvitamin D level, and no significant heterogeneity (I^2^ = 29.9%; *p* = 0.189) was observed between studies. Leave-out one study sensitivity analysis did not change the originally statistical significance of the pooling risk estimate. No evidence of publication bias was found according to the results of the Begg's test (*p* = 0.902), Egger's test (*p* = 0.428), and symmetrical funnel plot (Supplementary Figure S3).

### GRADE certainty of evidence

All-cause mortality was grouped as low quality, cardiovascular mortality was classified high quality, and MACEs was classified moderate quality (Supplementary Table S2).

## Discussion

The current meta-analysis focused on the predictive value of baseline 25-hydroxyvitamin D level in patients with CAD. This meta-analysis mainly found that 25-hydroxyvitamin D level at baseline was a significant predictor of MACEs, cardiovascular, and all-cause mortality in patients with CAD, even after adjusted multiple important confounders. Based on the comparison between the bottom and reference top 25-hydroxyvitamin D level, patients with the bottom 25-hydroxyvitamin D level conferred a 60 and 48%, higher risk of all-cause mortality and cardiovascular mortality, respectively. For the cardiovascular events, patients with the bottom category of 25-hydroxyvitamin D level had an approximately 33% higher risk of MACEs.

Several studies not meeting our criteria for inclusion also assessed the predictive value of 25-hydroxyvitamin D level in patients with CAD. The predictive role of 25-hydroxyvitamin D level was further supported *via* continuous variable analysis. In patients with stable angina pectoris, per 10 nmol/L decrease in 25-hydroxyvitamin D conferred a 9 and 10% higher risk of all-cause mortality and cardiovascular mortality, respectively (13). Based on Ludwigshafen Risk and Cardiovascular Health study, each standard deviation (SD) decrease in 25-hydroxyvitamin D level is associated with a 25% higher risk of all-cause mortality during 9.8 years follow-up in stable patients with CAD (24). Apart from the long-term outcomes, low 25-hydroxyvitamin D level is an independent predictor of cardiovascular mortality in patients with ACS (25). In patients with ST segment elevation myocardial infarction, low 25-hydroxyvitamin D level on admission is associated with high risk of no-reflow phenomenon (26).

The different types of CAD may affect the predictive value of 25-hydroxyvitamin D level. Based on subgroup analysis, the value of 25-hydroxyvitamin D level in the prediction of all-cause mortality was lower in patients with ACS (pooled RR 1.49) than in all CAD patients (pooled RR 1.64). Considering the lack of sufficient data, whether the predictive role of 25-hydroxyvitamin D level was affected by ACS subtypes was not determined. In addition, the predictive role of 25-hydroxyvitamin D level was weakened with the lengthening of follow-up in the subgroup analysis.

Several potential mechanisms may be implicated into the association of vitamin D deficiency with adverse outcomes in patients with CAD. First, low vitamin D can activate the activity of the renin-angiotensin-aldosterone system (27); Second, vitamin D deficiency may harm CAD patients by enhancing inflammation (28, 29). Finally, low 25-hydroxyvitamin D level was closely related to the occurrence of no-reflow phenomenon after percutaneous coronary intervention (26, 30) and the severity of CAD (31).

A recent meta-analysis of four randomized clinical trials suggested that vitamin D supplementation is associated with improvements in diastolic blood pressure and parathyroid hormone in patients with CAD having vitamin D deficiency (32). However, survival and cardiovascular events were not assessed in this meta-analysis. Based on our meta-analysis, CAD patients with low blood 25-hydroxyvitamin D level should be identified as high-risk group and be closely monitored. Future randomized controlled trials are required to demonstrate whether vitamin D supplementation could improve the prognosis of patients with CAD.

Several potential limitations should be addressed in this meta-analysis. Firstly, blood 25-hydroxyvitamin D level was only detected once rather than dynamic measurement, possibly causing classification bias. Secondly, the cut-off values of lower 25-hydroxyvitamin D level, which were used for predicting adverse outcomes, varied across studies, thus making it hard for clinicians to identify patients that need supplementation of vitamin D. Thirdly, significant heterogeneity was found for all-cause mortality. The different cut-off values of low 25-hydroxyvitamin D level, types of the CAD, or length of follow-up may contribute to the existing heterogeneity. Fourthly, this meta-analysis did not analyze the predictive role of 25-hydroxyvitamin D level by continuous data analysis because of the lack of sufficient data. Fifth, when a U-shaped association of 25-hydroxyvitamin D level with worse outcomes is observed (13, 33), the selection of the bottom 25-hydroxyvitamin D level as the reference may have led to underestimation of the actual risk summary. Finally, blood level of 25-hydroxyvitamin D is strongly correlated with time spent outdoors. The lack of adjusting season or time spent outdoors may have affected the pooling risk estimate.

## Conclusion

Low 25-hydroxyvitamin D level may be an independent predictor of MACEs, cardiovascular and all-cause mortality in patients with CAD. Baseline 25-hydroxyvitamin D level may provide important prognostic information in CAD patients.

## Data availability statement

The original contributions presented in the study are included in the article/Supplementary material, further inquiries can be directed to the corresponding authors.

## Author contributions

Study conception/design and interpretation of data: YF and XW. Literature search, data extraction, and quality assessment: HZ and PW. Statistical analysis: YJ and YS. Writing the manuscript: HZ. All the authors approved the version of the manuscript.

## Funding

This work is supported by (1) Suqian Science and Technology Support Project Fund (K201907), (2) Jiangsu 333 Talent Fund (BRA2020016), and (3) Zhenjiang Key Research and Development Fund (SH2021038).

## Conflict of interest

The authors declare that the research was conducted in the absence of any commercial or financial relationships that could be construed as a potential conflict of interest.

## Publisher's note

All claims expressed in this article are solely those of the authors and do not necessarily represent those of their affiliated organizations, or those of the publisher, the editors and the reviewers. Any product that may be evaluated in this article, or claim that may be made by its manufacturer, is not guaranteed or endorsed by the publisher.
