# Supplementary material for: Predictive value of 25-hydroxyvitamin D level in patients with coronary artery disease: A meta-analysis
Source: Front Nutr. 2022 Aug 10;9:984487. doi: 10.3389/fnut.2022.984487 (PMC9399797; doi:10.3389/fnut.2022.984487)
Supplement: Supplementary file 1 [file Data_Sheet_1.docx]

**Supplemental Text S1 – Search strategy**

**Databases---PubMed and Embase**

**Limits: Publications until** June 28, 2022

**1. PubMed Search**

Search: **((vitamin D OR 25-hydroxyvitamin D) AND (coronary artery disease OR coronary heart disease OR acute coronary syndrome OR myocardial infarction OR angina)) AND (death OR mortality OR cardiovascular event)** **486** items

| **2. Embase Search** | Query | Items found |
| --- | --- | --- |
| #1 | Search 'vitamin d'/exp OR 'vitamin d' OR (('vitamin'/expOR vitamin) AND d) | 367,636 |
| #2 | Search '25-hydroxyvitamin d'/exp OR '25-hydroxyvitamind' OR ('25 hydroxyvitamin' AND d) | 33,010 |
| #3 | Search #1 OR #2 | 368,017 |
| #4 | Search 'coronary artery disease'/exp OR 'coronary artery disease' OR (coronary AND ('artery'/exp OR artery) AND ('disease'/exp OR disease)) | 598,660 |
| #5 | Search 'coronary heart disease'/exp OR 'coronary heart disease' OR (coronary AND ('heart'/exp OR heart) AND ('disease'/exp OR disease)) | 994,577 |
| #6 | Search 'acute coronary' OR (acute AND coronary AND syndrome) | 103,628 |
| #7 | Search 'myocardial infarction'/exp OR 'myocardial infarction' OR (myocardial AND ('infarction'/exp OR infarction)) | 488,998 |
| #8 | Search 'angina'/exp OR angina | 131,029 |
| #9 | Search #4 OR #5 OR #6 OR #7 OR #8 | 1,127,312 |
| #10 | Search ('cardiovascular'/exp OR cardiovascular) AND event | 297,406 |
| #11 | Search 'mortality'/exp OR mortality | 1,781,017 |
| #12 | Search 'death'/exp OR death | 1,686,102 |
| #13 | Search #10 OR #11 OR #12 | 3,231,336 |
| #14 | Search 'follow up'/exp OR 'follow up' OR (follow AND up) | 2,354,471 |
| #15 | Search 'follow-up'/exp OR 'follow up' | 2,342,343 |
| #16 | Search #14 OR #15 | 2,354,471 |
| #17 | Search #3 AND #9 AND #13 AND #16 | 1,006 |
| #18 | #17 AND ('Article'/it OR 'Article in Press'/it) | **628** |
