# Supplementary material for: Predictive value of 25-hydroxyvitamin D level in patients with coronary artery disease: A meta-analysis
Source: Front Nutr. 2022 Aug 10;9:984487. doi: 10.3389/fnut.2022.984487 (PMC9399797; doi:10.3389/fnut.2022.984487)

Supplemental Figure S1 Funnel plot for all-cause mortality

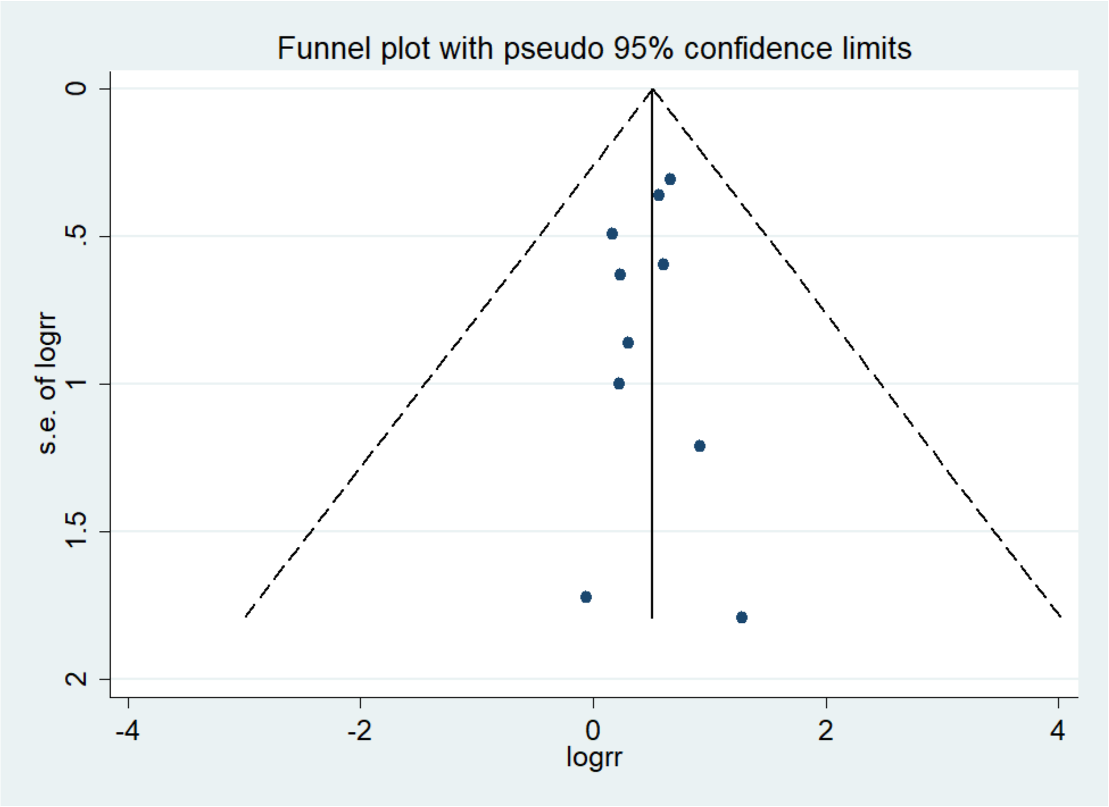

Supplemental Figure S2 Funnel plot for cardiovascular mortality

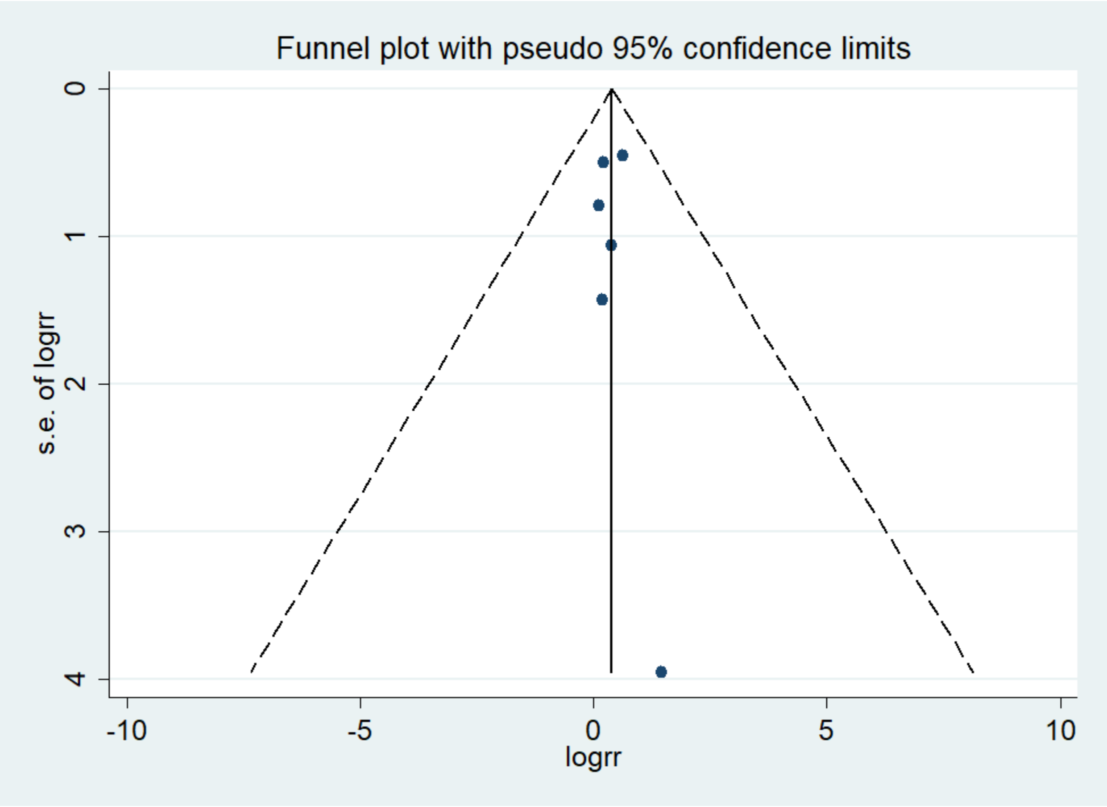

Supplemental Figure S3 Funnel plot for major adverse cardiovascular events

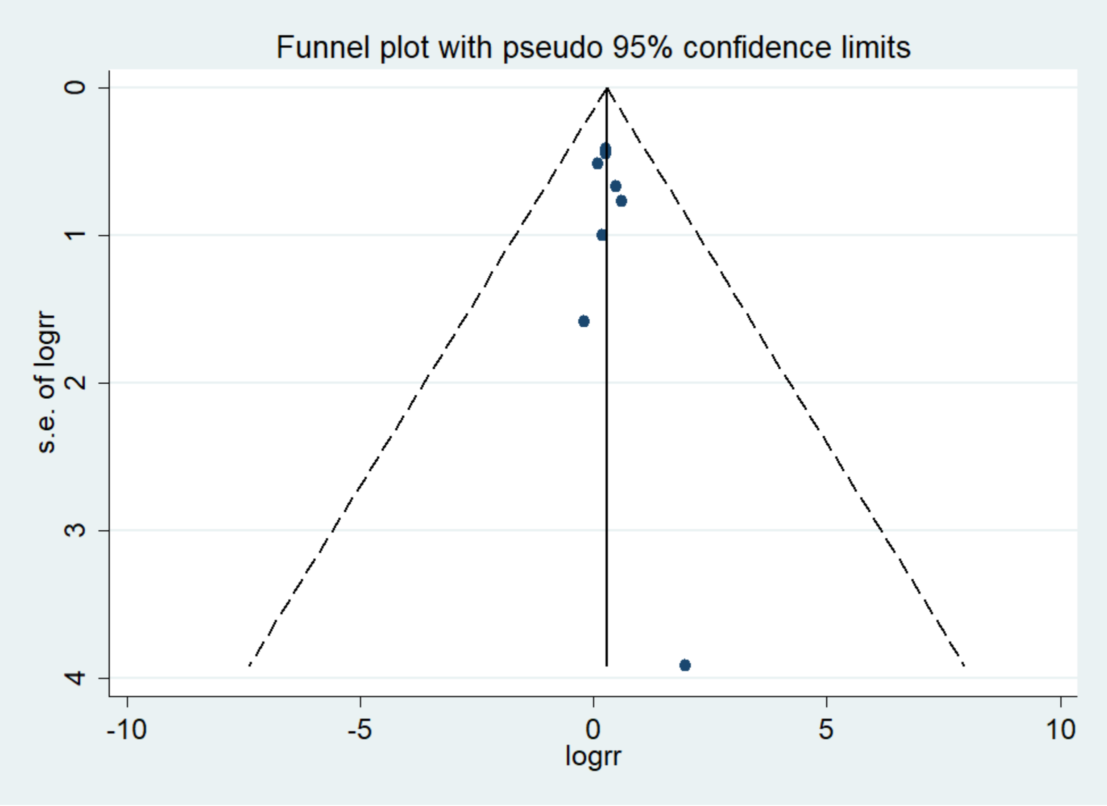

Supplement: Supplementary file 3 [file Presentation_1.pdf]
