# Supplementary material for: Predictive value of 25-hydroxyvitamin D level in patients with coronary artery disease: A meta-analysis
Source: Front Nutr. 2022 Aug 10;9:984487. doi: 10.3389/fnut.2022.984487 (PMC9399797; doi:10.3389/fnut.2022.984487)
Supplement: Supplementary file 4 [file Table_1.doc]

Supplemental Table S1 Methodological quality assessment using the Newcastle Ottawa Scale

| Author/year | Rrepresentativeness of the exposed cohort | Selection of the unexposed cohort | Ascertainment of exposure | Outcome of interest was not present at start of study | Comparability of cohorts on the design or analysis | Assessment of outcome | Was follow up long enough for outcomes to occur (>3 years) | Adequacy of follow-up of cohorts | Total scores |
| --- | --- | --- | --- | --- | --- | --- | --- | --- | --- |
| Grandi 2010 (14) |  | ★ | ★ | ★ | ★★ | ★ | ★ | ★ | 8 |
| Lerchbaum 2012 (9) | ★ | ★ | ★ | ★ | ★★ | ★ | ★ | ★ | 9 |
| Ng 2013 (10) |  | ★ | ★ | ★ | ★★ | ★ |  | ★ | 7 |
| Siasos 2013 (15) | ★ | ★ | ★ | ★ | ★★ |  |  | ★ | 7 |
| Welles 2014 (16) | ★ | ★ | ★ | ★ | ★★ |  | ★ | ★ | 8 |
| De Metrio 2015 (11) |  | ★ | ★ | ★ | ★★ | ★ |  | ★ | 7 |
| Naesgaard 2015 (17) |  | ★ | ★ | ★ | ★★ | ★ | ★ | ★ | 8 |
| Gerling 2016 (12) | ★ | ★ | ★ | ★ | ★★ | ★ | ★ | ★ | 9 |
| Yu 2018 (20) | ★ | ★ | ★ | ★ | ★★ | ★ | ★ | ★ | 9 |
| Degerud 2018 (13) |  | ★ | ★ | ★ | ★ | ★ | ★ | ★ | 7 |
| Beska 2019 (21) |  | ★ | ★ | ★ | ★★ | ★ |  | ★ | 7 |
| Aleksova 2020 (22) |  | ★ | ★ | ★ | ★★ | ★ |  | ★ | 7 |
| Verdoia 2021 (23) | ★ | ★ | ★ | ★ | ★★ |  |  | ★ | 7 |
