# Supplementary material for: Predictive value of 25-hydroxyvitamin D level in patients with coronary artery disease: A meta-analysis
Source: Front Nutr. 2022 Aug 10;9:984487. doi: 10.3389/fnut.2022.984487 (PMC9399797; doi:10.3389/fnut.2022.984487)
Supplement: Supplementary file 5 [file Table_2.docx]

Table S2 GRADE of evidence of outcomes of the included studies

| Outcomes |  | Certainty assessment | | | | | Effect | | Certainty | Importance |
| --- | --- | --- | --- | --- | --- | --- | --- | --- | --- | --- |
|  | No. of study | Risk of bias | Inconsistency | Indirectness | Imprecision | Publication bias | No. of patients | HR (95% CI) |  |  |
| All-cause mortality | 10 | Not serious | Serious | Serious | Not serious | Not serious | 16,265 | 1.60 (1.35 to 1.89) | ⊕⊕⊝⊝ **Low** | Critical |
| Cardiovascular mortality | 6 | Not serious | Not serious | Not serious | Not serious | Not serious | 10,092 | 1.48 (1.28 to 1.71) | ⊕⊕⊕⊕ **High** | Critical |
| MACEs | 7 | Not serious | Not serious | Not serious | Serious | Not serious | 6,476 | 1.33 (1.18 to 1.49) | ⊕⊕⊕⊝ **Moderate** | Important |

CI, confidence interval; HR, hazard ratio. MACEs, major adverse cardiovascular events; Risk of bias; no serious, study with Newcastle-Ottawa Scale ≥7 points; Inconsistency: Serious, I ^2^ > 50%; Imprecision (based on sample size): Serious, n < 10,000 participants.
